# Integrated multi-omics analysis reveals a lipid–redox regulatory network underlying drought tolerance and yield stability in rice

**DOI:** 10.1186/s12870-026-09098-1

**Published:** 2026-05-27

**Authors:** Rihan Hai, Xi Chen, Yanan Gao, Shan Lu, Xiaojing Wang, Xingjian Xu, Wanda Zhou, Rushun Duan, Guoxin Jiang, Jia Liu

**Affiliations:** 1 Key Laboratory of Soybean Molecular Design Breeding, State Key Laboratory of Black Soils Conservation and Utilization, Northeast Institute of Geography and Agroecology, Chinese Academy of Sciences, Harbin, 150000 China; 2 Key Laboratory of Rice Breeding Innovation in Northern Cold Region in Inner Mongolia, Hinggan League Academy of Agricultural and Animal Husbandry sciences, Ulanhot, 137400 China; 3 Jiamusi National Agricultural High Zone, Jiamusi, 156300 China; 4 Yazhouwan National Laboratory, Sanya, 572025 China

**Keywords:** Lipid metabolism, Redox homeostasis, Drought tolerance, Multi-omics analysis, Rice

## Abstract

**Background:**

Upland conditions in agroecosystems severely constrain rice productivity. To elucidate the molecular and physiological mechanisms underlying drought tolerance and yield stability in rice, integrated analyses of field phenotyping, transcriptomics, metabolomics, and whole-genome resequencing were conducted using two contrasting rice cultivars, DB46 and LK23.

**Results:**

Field evaluations showed that DB46 maintained superior yield stability under upland conditions, which was associated with increased leaf width, whereas LK23 exhibited pronounced leaf rolling that reduced transpiration but severely limited photosynthesis and biomass accumulation. Transcriptomic analyses revealed that DB46 coordinately up-regulated pathways related to fatty acid degradation, α-linolenic acid metabolism, and reactive oxygen species (ROS) detoxification, thereby supporting efficient energy mobilization and redox homeostasis. In contrast, these pathways were broadly suppressed in LK23, resulting in energy deficiency and oxidative stress. Metabolomic profiling further demonstrated significant enrichment of lipid- and secondary metabolism in DB46, including fatty acid biosynthesis, fatty acid metabolism, fatty acid elongation, fatty acid degradation, flavonoid biosynthesis, and phenylalanine metabolism, which collectively contributed to a robust antioxidant defense system. Whole-genome resequencing indicated a more conserved genomic architecture in DB46, whereas LK23 exhibited greater local polymorphism. Integrated multi-omics analyses identified 337 candidate genes associated with lipid metabolism and redox regulation, including lipoxygenase, protein phosphatase 1 L, and PP2C.

**Conclusions:**

Our findings uncover a lipid–redox regulatory network that underpins drought tolerance and yield stability in rice. These results provide valuable candidate genes and molecular targets for the genetic improvement and molecular design breeding of drought-resilient rice cultivars.

**Supplementary Information:**

The online version contains supplementary material available at 10.1186/s12870-026-09098-1.

## Introduction

Rice (*Oryza sativa L.*) is one of the most important staple crops worldwide, serving as the primary food source for more than half of the global population and playing a central role in global food security. It is cultivated on more than 160 million hectares globally, and its yield and grain quality directly determine the stability and sustainability of the world’s food supply [1]. Conventional rice cultivation relies heavily on adequate irrigation, and the flooded paddy system has been historically effective in maintaining high productivity and grain quality [2, 3]. However, the increasing severity of climate change, the frequent occurrence of drought events, and the growing scarcity of freshwater resources have collectively posed serious challenges to the sustainability of rice production systems [4–6]. Currently, more than one-quarter of the global rice-growing area is affected by varying degrees of water limitation, particularly in South Asia, Southeast Asia, and northern China, where insufficient water availability has become a major constraint to stable rice yields. Under these circumstances, the sustainability of traditional irrigated rice systems is being increasingly questioned, and the development of water-saving and drought-adaptive rice varieties has become a critical direction for future rice breeding [7–9]. Upland rice, which is cultivated under rainfed or non-flooded conditions, represents a valuable genetic resource for drought adaptation. It possesses several adaptive traits, including deeper and more extensive root systems, improved water use efficiency, and higher photosynthetic tolerance to water deficit [10–12]. However, the yield and grain quality of upland rice are generally lower than those of lowland rice, reflecting a typical trade-off between drought tolerance and productivity [13, 14]. Therefore, elucidating the physiological and molecular mechanisms underlying drought adaptation in upland rice is of great scientific and practical importance for achieving a balance between yield potential and drought resilience.

Drought stress is one of the major abiotic factors limiting rice yield formation and grain quality, and its regulatory process involves complex physiological, biochemical, and molecular networks. Accordingly, drought response has long been a central topic in rice stress biology. Classical theory categorizes rice drought-adaptive strategies into three types: escape, avoidance, and tolerance. Escape strategies rely on developmental adjustments that enable plants to bypass periods of severe drought; avoidance strategies enhance water acquisition and conservation through improved root growth, regulation of stomatal aperture, and maintenance of cellular osmotic balance; whereas tolerance strategies mitigate cellular damage by strengthening antioxidant capacity, accumulating osmoprotectants, and stabilizing membrane structures [15–18]. Root architectural traits represent a critical component of drought avoidance. Drought-tolerant rice varieties typically develop deeper, thicker, and denser root systems, enabling more efficient absorption of water from deeper soil layers. Overexpression of *DRO1* significantly enhances root downward growth, thereby improving water-use efficiency (WUE) and stabilizing yield under drought conditions [19] In addition, *Robust Root System 1* (*RRS1*) negatively regulates root development by promoting the expression of *OsIAA3*, thus modulating auxin signaling and contributing to drought-responsive root morphological adjustments [20]. Beyond root adaptation, stomatal regulation, osmotic adjustment, and redox homeostasis constitute core mechanisms of drought tolerance. *OsSLAC1* enhances drought resistance by optimizing stomatal closure and reducing transpirational water loss [21]. In terms of osmotic regulation, *OsP5CS* promotes proline biosynthesis, which helps maintain cellular osmotic potential and alleviates dehydration stress [22]. Meanwhile, activation of OsAPX2 effectively scavenges reactive oxygen species (ROS), maintaining redox balance and reducing oxidative damage [23]. Moreover, the gibberellin signaling repressor SLR1 enhances drought tolerance by negatively regulating the abundance of the dehydration-responsive protein OsBURP3 [24].

Lipid metabolism encompasses the synthesis, degradation, and remodeling of fatty acids, glycerides, phospholipids, sphingolipids, and other lipid species within plants. It not only contributes to the maintenance of cellular membrane structure, energy storage, and signal transduction, but also plays a pivotal role in plant responses to abiotic stresses, such as cold, salinity, and heat, as well as in developmental processes, including pollen maturation and grain filling [25–27]. Lipid metabolism regulates membrane fluidity, antioxidant capacity, and metabolic homeostasis, thereby serving as a critical physiological mechanism for plants to adapt to environmental changes and ensure yield formation [28]. Previous studies have shown that under cold stress, the membrane lipid composition and fatty acid unsaturation of rice leaves undergo significant alterations, with remodeling of phospholipids (e.g., PC, PE, PG) and glycerolipids (e.g., MGDG, DGDG) maintaining membrane fluidity and chloroplast function, thereby safeguarding photosynthetic efficiency and leaf cold tolerance. ALA10 interacts with the fatty acid desaturase FAD2 to modulate the fatty acid composition and degree of unsaturation of phosphatidylcholine (PC), thereby enhancing plant adaptation to low-temperature conditions [29–31]. Under salinity stress, membrane lipids in rice roots, including PA, PC, and PS, as well as sphingolipids such as Cer and SPH, are markedly upregulated, accompanied by differential expression of lipid metabolism-related genes; this regulation enhances membrane stability, osmotic adjustment, and root tolerance to ion toxicity [32]. Heat stress also strongly affects lipid metabolism; for instance, the *HTS1* gene, encoding β-ketoacyl carrier protein reductase (KAR) involved in fatty acid biosynthesis, is essential for maintaining proper membrane lipid composition, and its loss disrupts membrane integrity and reduces thermotolerance [33]. Beyond stress responses, lipid metabolism is critical during reproductive development and grain filling, with lipid transporters and phospholipid synthases contributing to pollen maturation, anther cuticle formation, and storage lipid accumulation in grains, thereby influencing grain quality and yield formation [4, 34]. However, given the critical roles of lipid metabolism in membrane homeostasis, signaling regulation, and abiotic stress adaptation, its functional mechanisms in rice drought response and yield stability remain to be thoroughly elucidated.

In this study, two rice varieties with contrasting drought tolerance were cultivated under both paddy and upland field conditions, rather than relying on laboratory-simulated drought, to better reflect responses under realistic agricultural production. By integrating untargeted metabolomics, transcriptomics, and genomics data, we systematically investigated the role of root and leaf lipid metabolism in maintaining stable rice yield. Our results demonstrated that the drought-tolerant variety could coordinate root and leaf lipid metabolism under upland conditions to maintain a “source–sink” balance, thereby sustaining yield, whereas the sensitive variety exhibited a disruption in this mechanism, leading to yield reduction. These findings clearly represent a tolerance strategy within the three classical drought-response strategies in rice, highlighting the central role of lipid metabolism in maintaining cellular homeostasis and yield stability. This study not only advances our understanding of lipid metabolism function under upland cultivation but also provides a molecular basis for breeding high-yielding drought-tolerant rice based on the tolerance strategy.

## Materials and methods

### Plant materials and sample collection

Based on preliminary drought-resistance screening of 300 rice accessions at the seedling stage, a drought-tolerant and a drought-sensitive conventional *japonica* rice variety, DB46 and LK23, respectively, were selected for a field-based comparative study. The experiment was conducted in a single ecological region of Hinggan League on loamy black soil. To capture natural water-deficit responses, plants were cultivated under two contrasting, realistic field conditions: conventional paddy fields with 5–10 cm water layer and regular NPK fertilization (120 kg N, 60 kg P₂O₅, 80 kg K₂O ha⁻¹; irrigation every 7 days) and upland fields on adjacent elevated land relying solely on natural rainfall, with drainage ditches to prevent waterlogging and no supplemental irrigation. Both treatments shared consistent soil type and field management practices, including weed and pest control. A completely randomized block design with three biological replicates per treatment was employed, with each plot covering 20 m² (row spacing 25 cm; plant spacing 15 cm). At 10 days after the onset of grain filling (D10), the third leaf and 2 cm segment of the primary root were collected from five randomly selected plants per plot, with three biological replicates; samples were pooled, immediately frozen in liquid nitrogen, and stored at − 80 °C for subsequent metabolomic and transcriptomic analyses.

### RNA extraction and transcriptome sequencing

Total RNA was extracted from each sample using the RNAprep Pure Kit (Tiangen, Beijing, China). Only high-quality RNA (OD260/280 = 1.8–2.2, RIN ≥ 7) was used to construct mRNA libraries. The libraries were sequenced on an Illumina NovaSeq 6000 platform in paired-end 150 bp (PE150) mode. Raw reads were filtered to remove adapters, low-quality sequences, and contaminants. Clean reads were aligned to the rice reference genome (ncbi_oryza_sativa_japonica_group_gca_009797565_1_osativa_kitaake_v2_0) using HISAT2. Gene expression abundance (FPKM) was estimated using Kallisto, and differential expression analysis based on count data, including the calculation of P-values and fold changes, was performed using DESeq2 with thresholds of a false discovery rate (FDR) < 0.05 and fold change (FC) ≥ 2 or ≤ 0.5.

### Metabolite profiling

Metabolomic analysis was performed using a Waters Acquity IClass PLUS UPLC system coupled with a Xevo G2-XS QTOF mass spectrometer. Samples were separated on a Waters Acquity UPLC HSS T3 column (1.8 μm, 2.1 × 100 mm) with a mobile phase consisting of 0.1% formic acid in water (solvent A) and acetonitrile (solvent B). Injections of 1 µL were analyzed in both positive and negative ionization modes. The mass spectrometer was operated in MSe mode (MassLynx V4.2) with a collision energy ramp of 2–40 V and an acquisition rate of 0.2 s/spectrum. The ion source parameters were as follows: capillary voltage of ± 2000/1500 V, cone voltage of 30 V, source temperature of 150 °C, and desolvation gas flow of 800 L/h. Raw data were processed using Progenesis QI software, and metabolite identification and quantification were performed based on the METLIN and Biomark in-house databases, with a mass deviation of less than 100 ppm. For statistical analyses, principal component analysis (PCA), Spearman correlation analysis, and orthogonal partial least squares discriminant analysis (OPLS-DA; v.14.1, Umetrics, Umeå, Sweden) were employed to reveal metabolic variations among samples. The OPLS-DA model was validated using 200 permutation tests implemented in the *ropls* R package, and variable importance in projection (VIP) values were extracted for all metabolites. Differential metabolites were identified based on integrated criteria: VIP ≥ 1, fold change (FC) ≥ 2 or ≤ 1, and statistical significance (*P* < 0.05, FDR < 0.05). Finally, the identified differential metabolites were subjected to pathway enrichment analysis using hypergeometric testing in the KEGG and LipidMaps databases.

### Whole-genome resequencing and variant detection

For the two rice varieties, DB46 and LK23, leaf tissue was collected from five healthy plants, pooled, immediately frozen in liquid nitrogen, and stored at − 80 °C until use. Total genomic DNA was extracted using the Plant Genomic DNA Kit (Tiangen, Beijing, China) according to the manufacturer’s instructions. Approximately 10 mg of genomic DNA per sample was used to construct paired-end sequencing libraries with an insert size of 350 bp. The libraries were sequenced by Novogene Co., Ltd. using a whole-genome resequencing approach on the Illumina NovaSeq 6000 platform, generating 150 bp paired-end (PE150) reads, with a sequencing depth exceeding 20×coverage for each sample. Raw sequencing reads were quality-filtered using Trimmomatic (Bolger et al., 2014, ver. 0.39) with parameters of Phred score ≥ 20 and read length ≥ 30 bp to obtain clean reads. Clean reads were aligned to the rice reference genome (https://rapdb.dna.affrc.go.jp) using BWA mem (2010, ver. 0.7.17). The resulting alignments were converted to BAM files with SAMtools (ver. 1.7), and duplicate reads were removed using Picard (MarkDuplicates, ver. 2.18.9). SNPs and InDels were called using GATK (ver. 3.8.0), and genome coverage was estimated with bamdst (ver. 1.0.9). Variants were stringently filtered: SNPs with QD ≥ 2, MQ ≥ 40, FS ≤ 60, SOR ≤ 3, MQRankSum ≥ − 12.5, ReadPosRankSum ≥ − 8; InDels with QD ≥ 2, FS ≤ 200, SOR ≤ 10, MQRankSum ≥ − 12.5, ReadPosRankSum ≥ − 8; and all variants required to have two alleles, missing rate ≤ 0.9, minor allele frequency ≥ 0.05, and mean depth ≥ 5. Filtered SNPs were annotated with ANNOVAR (ver. 2015 Dec 14) based on the rice reference genome, and mutation analysis and visualization were performed using the R package maftools.

### qRT-PCR validation

To investigate the expression patterns of differentially expressed genes (DEGs) in rice varieties DB46 and LK23 under paddy and upland field conditions, qRT-PCR validation was performed. During the grain-filling stage, leaf and root tissues were collected from both varieties under the two cultivation environments for analysis. Total RNA was extracted using a magnetic bead–based method (NanoMagbio), and first-strand cDNA was synthesized using the ReverTra Ace™ qPCR RT Master Mix (TOYOBO). Quantitative real-time PCR (qRT-PCR) was performed using SYBR Green PCR Master Mix (TRADEKEY) on a QuantStudio™ 5 Real-Time PCR System (Thermo Fisher Scientific). Primers were designed using Primer Premier 5 software. The *Ubiquitin1* gene (*LOC_Os03g50885*) was employed as the internal reference, and relative expression levels were calculated using the 2^–ΔΔCT method. Three biological replicates were included for each sample. Statistical analysis was performed using Student’s *t*-test or one-way ANOVA, and differences were considered significant at *P* < 0.05. Primer sequences are listed in Table S4.

## Results

### Phenotypic assessment under field conditions

**Fig. 1:**
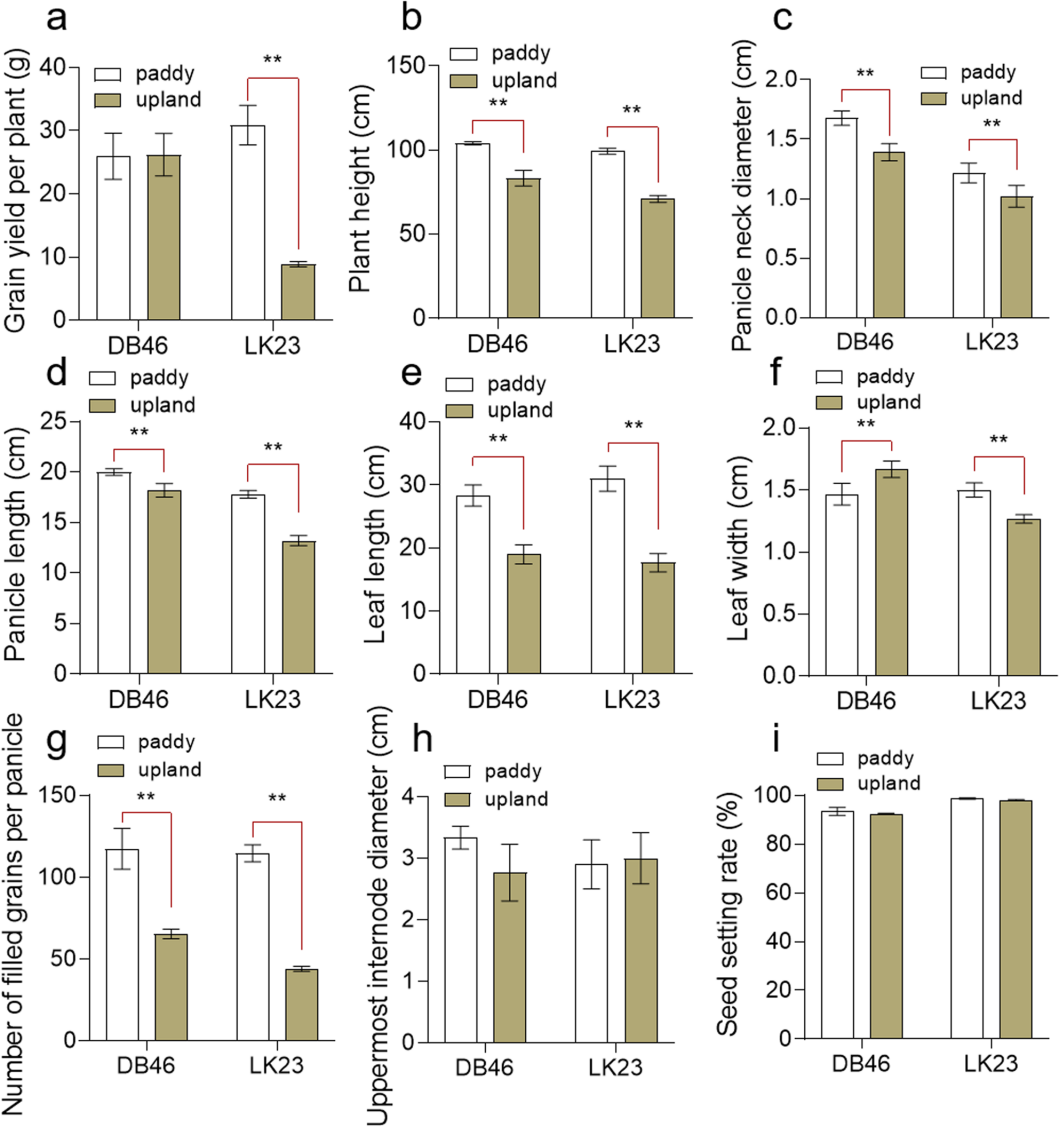
Comparison of major agronomic traits between rice varieties DB46 and LK23 under paddy and upland conditions. **a** Grain yield per plant. **b** Plant height. **c** Panicle neck diameter. **d** Panicle length. **e** Leaf length. **f** Leaf width. **g** Number of filled grains per panicle. **h** Uppermost internode diameter. **i** Seed setting rate, asterisks indicate statistical significance by two-tailed Student’s *t* tests (*P < 0.05, **P < 0.01)

To validate the drought tolerance of the two genotypes under field conditions and to assess their phenotypic responses to water availability, both genotypes were cultivated under paddy and upland conditions for comparative analysis. In a preliminary laboratory screening of 200 rice accessions under drought stress, two representative genotypes with contrasting drought tolerance were identified—LK23 (drought-sensitive) and DB46 (drought-tolerant). The results demonstrated that DB46 exhibited significantly greater drought tolerance than LK23 (Fig. 1). Agronomic traits, including yield per plant, plant height, panicle length, leaf length, leaf width, panicle–stem thickness, and seed-setting rate, were measured to evaluate the drought-induced phenotypic variations. Under upland conditions, LK23 showed a pronounced reduction in multiple traits—such as plant height, panicle length, leaf length, grain number per panicle, and stem thickness—whereas DB46 was much less affected. Notably, leaf width increased significantly in DB46 but decreased in LK23, suggesting distinct morphological adaptations to drought. Most importantly, under upland conditions, the yield per plant of LK23 decreased significantly, whereas that of DB46 remained stable (Figure S1).

### Transcriptome analysis and clustering of differentially expressed genes under different cultivation conditions in DB46 and LK23

**Fig. 2:**
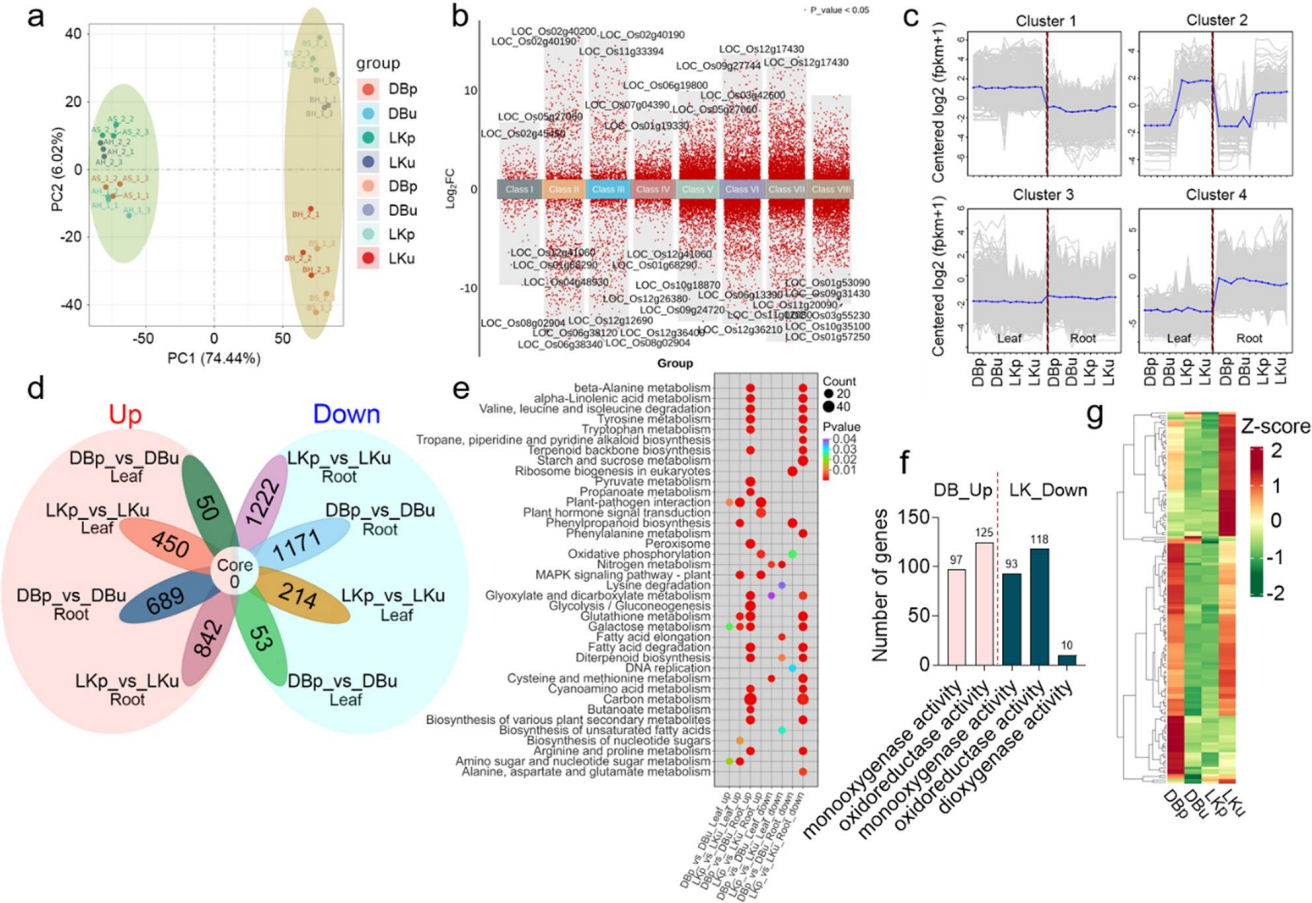
Transcriptomic landscape and differential expression analysis of rice cultivars DB46 and LK23 under paddy and upland conditions. DB46 (DB) and LK23 (LK) under paddy (p) and upland (u) conditions. **a** Principal component analysis (PCA) of RNA-seq samples from leaves and roots. **b** Distribution of Log2 fold changes (Log2FC) for Differentially expressed genes (DEGs) in distinct comparison groups, with the most significantly altered gene IDs highlighted. Class I–VIII represent the distribution of differentially expressed genes between the two groups, corresponding respectively to DBp_vs_DBu_Leaf, DBp_vs_LKp_Leaf, DBu_vs_LKu_Leaf, LKp_vs_LKu_Leaf, DBp_vs_DBu_Root, DBp_vs_LKp_Root, DBu_vs_LKu_Root, and LKp_vs_LKu_Root. **c** K-means clustering of gene expression patterns. Gray lines represent the expression trends of individual genes, while blue lines indicate the mean expression profile of each cluster across leaf and root samples from the four treatment groups (DBp, DBu, LKp, LKu). **d** Flower plot showing the numbers of upregulated (left, red/pink petals) and downregulated (right, blue/green petals) DEGs across different comparison groups. **e** KEGG pathway enrichment bubble plot of DEGs. Bubble size represents the number of enriched genes, and color gradients indicate the level of statistical significance (*P* value). **f** Statistics of DEGs enriched in specific GO molecular functions, including monooxygenase and oxidoreductase activities. The bar chart compares the number of upregulated genes in DB46 under upland conditions (DB_Up) with downregulated genes in LK23 (LK_Down). **g** Hierarchical clustering heatmap of selected key candidate genes. The color scale represents Z-score–standardized FPKM values (red indicates high expression and green indicates low expression)

To investigate the gene expression response strategies of DB46 and LK23 under upland conditions, RNA-seq analysis was performed on leaf and root tissues of both genotypes grown under paddy and upland cultivation conditions. A total of 164.32 Gb of clean data were obtained from 24 samples. After quality control, all samples exhibited Q30 values above 96.75%, with an average alignment efficiency of 91.88% (Table S1), indicating high data quality. Principal component analysis (PCA) revealed strong consistency among biological replicates and clear separation between aboveground and belowground tissues (Fig. 2a). Among different cultivation environments and tissue types, the roots exhibited the highest number of differentially expressed genes (DEGs), whereas the leaves displayed the fewest (Fig. 2b). To identify differential expression patterns across different tissues and genotypes, all differentially expressed genes were subjected to k-means clustering to group genes with similar expression trends. Four major expression pattern groups were obtained (Fig. 2c). Interestingly, regardless of cultivation conditions, these four clusters exhibited distinct tissue- and genotype-specific expression characteristics. In Cluster 1, 2,331 differentially expressed genes were significantly up-regulated in the leaves of both genotypes but down-regulated in the roots. In Cluster 2, 229 genes were up-regulated in both leaves and roots of LK23, whereas the same genes were down-regulated in DB46. Cluster 3 contained 6,245 differentially expressed genes that were generally down-regulated in both leaves and roots of both genotypes. In Cluster 4, 2,302 genes were down-regulated in leaves but up-regulated in roots. Collectively, these findings indicate that, in DB46 and LK23, the expression of a subset of genes is primarily governed by intrinsic regulatory mechanisms associated with tissue type and genotype rather than by cultivation conditions, suggesting that tissue specificity and genetic background play dominant roles in transcriptional regulation.

To further identify key genes responsive to environmental differences, differential expression analyses were performed for DB46 and LK23 across different cultivation conditions and tissue types (Fig. 2d). In DB46, 50 and 689 genes were specifically up-regulated in leaves and roots, respectively, under upland conditions, while 53 and 1,171 genes were down-regulated. In contrast, in LK23, 450 and 842 genes were specifically up-regulated in leaves and roots, respectively, under upland conditions, whereas 214 and 1,222 genes were down-regulated. Based on the KEGG enrichment analysis of differentially expressed genes (DEGs) between paddy and upland conditions (Fig. 2e), the DEGs in DB46 leaves were significantly enriched in metabolic pathways such as galactose metabolism, nitrogen metabolism, and glyoxylate and dicarboxylate metabolism. In contrast, the DEGs in LK23 leaves were mainly enriched in biosynthesis of nucleotide sugars and phenylpropanoid biosynthesis pathways. Further analysis revealed that the up-regulated genes in DB46 roots were significantly enriched in carbon metabolism, fatty acid degradation, peroxisome, alpha-linolenic acid metabolism, tryptophan metabolism, and tyrosine metabolism. Conversely, these enriched pathways corresponded primarily to the down-regulated genes in LK23 roots, indicating that the two varieties exhibit opposite regulatory trends in these metabolic processes.

Gene Ontology (GO) enrichment analysis revealed that the differentially expressed genes (DEGs) exhibited significant changes in enrichment across molecular function, cellular component, and biological process categories, with the most prominent differences observed in molecular functions (Figure S2). In the roots of DB46, genes associated with monooxygenase activity and oxidoreductase activity were significantly up-regulated, comprising 97 and 125 genes, respectively. In contrast, in the roots of LK23, the corresponding genes were significantly down-regulated, totaling 93 and 118 genes, respectively. Additionally, ten down-regulated genes associated with dioxygenase activity were identified in LK23 (Fig. 2f). Heatmap analysis further indicated that these genes exhibited opposite expression trends between the two varieties in response to cultivation conditions (Fig. 2g). Collectively, these results suggest that oxidation–reduction reactions related to lipid metabolism play a crucial role in the adaptive responses of DB46 and LK23 to different cultivation environments.

### Metabolomic profiling and pathway analysis of drought responses in LK23 and DB46

**Fig. 3:**
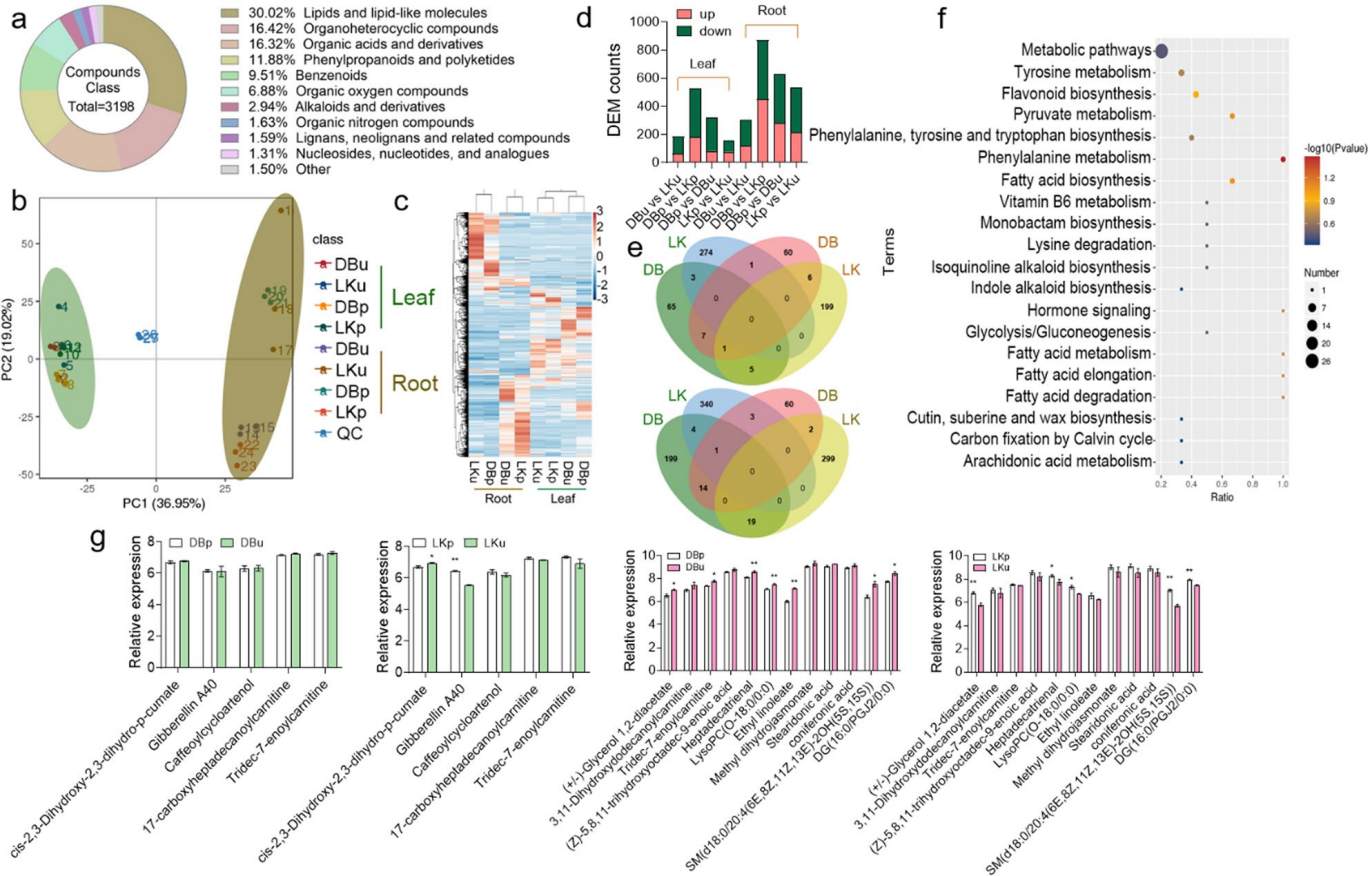
Metabolomic profiling and analysis of Differentially accumulated metabolites (DAMs) in rice varieties DB46 and LK23 under paddy and upland conditions. **a** Classification of total identified metabolites. The pie chart displays the proportions of different chemical classes among the 3,198 detected compounds. **b** Principal Component Analysis (PCA) score plot of metabolite profiles derived from leaf (green region) and root (brown region) samples. **c** Hierarchical clustering heatmap of metabolite abundances. The color scale represents row-scaled Z-scores, with red indicating high abundance and blue indicating low abundance. **d** Number of up-regulated (red bars) and down-regulated (green bars) DAMs in pairwise comparisons across leaf and root tissues. **e** Venn diagrams illustrating the overlap of DAMs between different comparison groups. **f** KEGG pathway enrichment analysis of DAMs. The dot size represents the number of enriched metabolites, and the color gradient indicates the significance level (-log10 P-value). **g** Relative abundances of lipids and lipid-like molecules involved in the drought response. Upper panels (green bars) and lower panels (pink bars) represent data from leaves and roots, respectively, asterisks indicate statistical significance by two-tailed Student’s *t* tests (*P < 0.05, **P < 0.01)

To validate the metabolic responses associated with the identified key genes and to elucidate the response strategies of related metabolites, a comprehensive metabolomic analysis was conducted on LK23 and DB46 under different environmental conditions. A total of 3,198 metabolites were identified and classified into major chemical categories based on their structural characteristics (Fig. 3a). Among them, lipids and lipid-like molecules accounted for the largest proportion (30.02%), indicating that lipid metabolism plays a predominant role in the samples and is likely associated with membrane stability, energy storage, and stress signal transduction. Organoheterocyclic compounds (16.42%) and organic acids and derivatives (16.32%) ranked next, suggesting active primary and secondary metabolism. Phenylpropanoids and polyketides (11.88%) and benzenoids (9.51%) were also abundant, mainly involved in the biosynthesis of aromatic compounds, pigments, and defense-related metabolites. In addition, organic oxygen compounds (6.88%), alkaloids and derivatives (2.94%), and organic nitrogen compounds (1.63%) were detected, while minor categories included lignans and neolignans (1.59%), nucleosides and nucleotides (1.31%), and others (1.50%). Principal component analysis (PCA) of the global metabolomic data (Fig. 3b) revealed a clear separation between tissue types along the first two principal components, with leaf and root samples distinctly clustered, indicating a strong tissue-specific effect on metabolite composition. Further examination showed that leaf samples from both genotypes grown under paddy and upland conditions clustered closely, suggesting relatively stable metabolic profiles in leaves across environments. In contrast, root samples exhibited pronounced spatial separation, implying that root metabolism is more sensitive to environmental variation and contributes substantially to the overall metabolic divergence between cultivation conditions. Statistical analyses identified 1,735 differentially expressed metabolites (DEMs) across tissues, genotypes, and environments. Hierarchical clustering of metabolite abundances (Fig. 3c) revealed clear segregation between root and leaf tissues, as well as distinct clustering between paddy and upland conditions. These findings indicate that both tissue type and cultivation environment jointly shape the metabolic landscape, serving as major determinants of metabolic variation.

Among the DEMs (Fig. 3d), the number of downregulated metabolites generally exceeded that of upregulated ones, particularly in roots, where the magnitude of metabolic change was greater than in leaves. This suggests that root metabolism exhibits stronger plasticity and regulatory capacity in response to environmental stress. Venn analysis of up- and downregulated metabolites across genotypes and tissues (Fig. 3e) further demonstrated limited overlap among groups, reflecting pronounced tissue specificity and genotypic variation in drought responses. Specifically, the numbers of uniquely upregulated metabolites were 274 and 65 in the leaves of LK23 and DB46, and 199 and 60 in the roots, respectively; for downregulated metabolites, the corresponding numbers were 340 and 199 in leaves and 299 and 60 in roots. Overall, both genotypes exhibited distinct metabolic adjustment patterns, with the drought-sensitive LK23 showing more extensive metabolic alterations, indicating a stronger yet less efficient metabolic response to environmental changes, likely contributing to its greater yield reduction under drought. KEGG enrichment analysis of the differential metabolites in roots revealed significant enrichment in fatty acid biosynthesis, fatty acid metabolism, fatty acid degradation, phenylalanine metabolism, and flavonoid biosynthesis pathways (Fig. 3f, S2). Notably, lipid- and secondary-metabolism–related pathways were more strongly enriched in the drought-tolerant DB46 under upland cultivation, suggesting that enhanced lipid remodeling and secondary metabolism contribute to its superior drought adaptation and yield stability. In addition, five and twelve metabolites involved in lipid metabolism and its derivative pathways were identified in leaves and roots, respectively. Interestingly, in leaves, lipid-related metabolites in the drought-tolerant DB46 remained largely unchanged under upland conditions, while they were significantly reduced in the sensitive LK23. Conversely, in roots, these twelve lipid-associated metabolites were markedly decreased in LK23 but significantly accumulated in DB46 under upland conditions.

### Integrated transcriptomic and metabolomic analyses reveal distinct drought-response mechanisms in rice genotypes

**Fig. 4:**
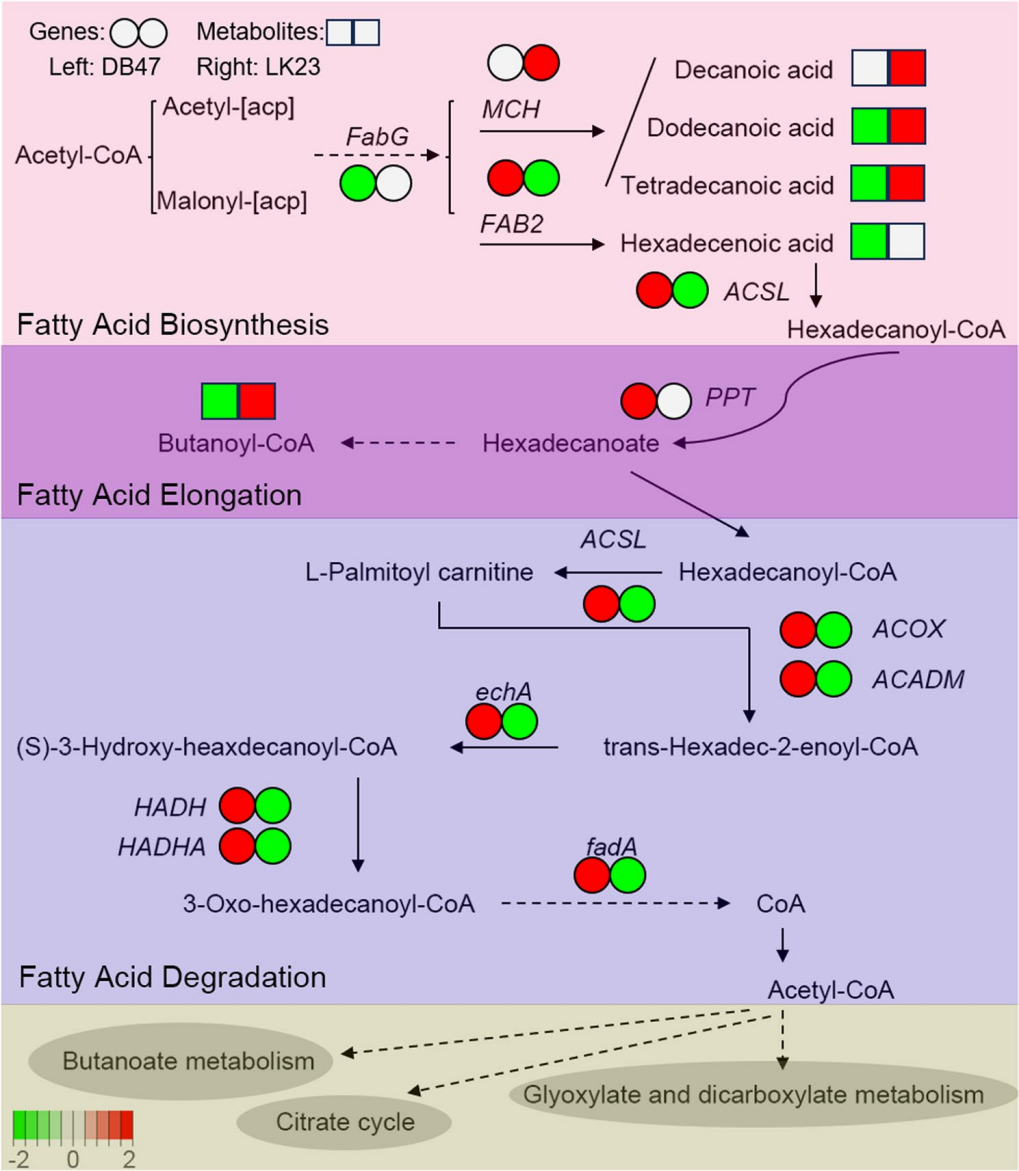
Integrated transcriptomic and metabolomic analysis of fatty acid metabolism pathways in rice varieties DB46 and LK23. The schematic diagram illustrates metabolic alterations across the “Fatty Acid Biosynthesis”, “Fatty Acid Elongation”, and “Fatty Acid Degradation” pathways. Circles and rectangles represent Differentially expressed genes (DEGs) and Differentially accumulated metabolites (DAMs), respectively. Each node is divided into two sections: the left half represents the expression or accumulation pattern in DB46, while the right half corresponds to LK23. The color scale indicates the Log2 fold change (Log2FC) relative to the control, ranging from green (down-regulation) to red (up-regulation), with white indicating no significant change. Key genes (e.g., *FabG*, *FAB2*, *ACSL*, *ACOX*) and metabolites (e.g., decanoic acid, hexadecanoic acid) are mapped to their respective catalytic steps. Dashed arrows denote multi-step reactions or indirect linkages to other metabolic pathways, including the citrate cycle and butanoate metabolism

To explore the correlation between differentially expressed genes (DEGs) and differentially accumulated metabolites (DAMs) in the roots of drought-sensitive and drought-tolerant rice varieties under upland conditions, an integrated analysis of transcriptomic and metabolomic data was performed. KEGG enrichment analysis revealed that multiple metabolic pathways were co-enriched in both DEGs and DAMs. Among them, the fatty acid biosynthesis (map00061), fatty acid elongation (map00062), and fatty acid degradation pathways were significantly enriched (*p* < 0.05). In addition, several secondary metabolite biosynthesis pathways, including phenylalanine metabolism (map00360), diterpenoid biosynthesis (map00904), flavonoid biosynthesis (map00941), and phenylpropanoid biosynthesis (map00940), were also highly enriched (Figure S3). To further elucidate the regulatory role of fatty acid metabolism in rice roots responding to upland conditions, a metabolic pathway map was constructed based on the associations between DEGs and DAMs. The analysis showed that under drought conditions, the drought-tolerant (DB46) and drought-sensitive (LK23) varieties exhibited markedly distinct metabolic fluxes and gene expression patterns within fatty acid synthesis, elongation, and degradation pathways (Fig. 4).

In the fatty acid biosynthesis and elongation modules, metabolomic profiling indicated that the accumulation of specific free fatty acids and acyl-CoA intermediates was markedly suppressed in LK23 under upland conditions. Specifically, compared with DB46, the levels of decanoic acid, dodecanoic acid, tetradecanoic acid, and butanoyl-CoA failed to accumulate effectively in LK23, whereas these metabolites were significantly increased in DB46. At the transcriptional level, ACSL (long-chain acyl-CoA synthetase) and PPT genes showed significantly lower expression in DB46, accompanied by a notable accumulation of free fatty acids. This suggests that DB46 tends to enhance fatty acid processing and utilization rather than de novo synthesis under drought conditions, thereby maintaining membrane stability and improving drought tolerance. In contrast, LK23 exhibited an opposite pattern, with elevated expression of biosynthetic genes but reduced metabolite accumulation, indicating an impaired capacity for fatty acid processing and downstream utilization, which may restrict its adaptive response to drought. A similar pattern was observed in the fatty acid elongation pathway, where butanoyl-CoA was significantly accumulated in the drought-tolerant DB46. Moreover, transcriptomic profiling revealed a distinct regulatory pattern in the fatty acid degradation pathway. Under upland conditions, DB46 appeared to fully activate the fatty acid β-oxidation pathway. A series of key enzyme genes involved in oxidation, hydration, and dehydrogenation processes, including *ACSL*, *ACOX*, *ACADM*, *echA*, *HADH*, *HADHA*, and *fadA*, consistently exhibited lower expression levels in DB46, corresponding to enhanced metabolite accumulation and efficient lipid mobilization. Conversely, in LK23, the entire gene cluster showed an overall upregulation trend without corresponding metabolic enhancement, suggesting an inefficient transcription–metabolism coupling and a reduced capacity for lipid degradation and energy redistribution under upland conditions. These findings demonstrate that key enzyme genes involved in oxidation, hydration, and dehydrogenation processes exhibit significant expression differences between DB46 and LK23, thereby verifying the pivotal regulatory role of lipid metabolism in upland conditions responses.

### Comparative genotypic profiling of DB46 and LK23 through whole-genome resequencing

To comprehensively characterize the genetic diversity between DB46 and LK23 and to systematically identify genomic variants, we performed deep whole-genome resequencing of both cultivars, generating 18.54 Gb of high-quality clean data (Q30 ≥ 96.44%) with an average sequencing depth of 20× per sample. After alignment of the sequencing reads to the rice reference genome, we identified 176,950 high-quality single-nucleotide polymorphisms (SNPs) and 46,850 insertions/deletions (InDels) in DB46, and 271,110 high-quality SNPs and 65,868 InDels in LK23 (Supplementary Tables 2 and 3).

**Fig. 5:**
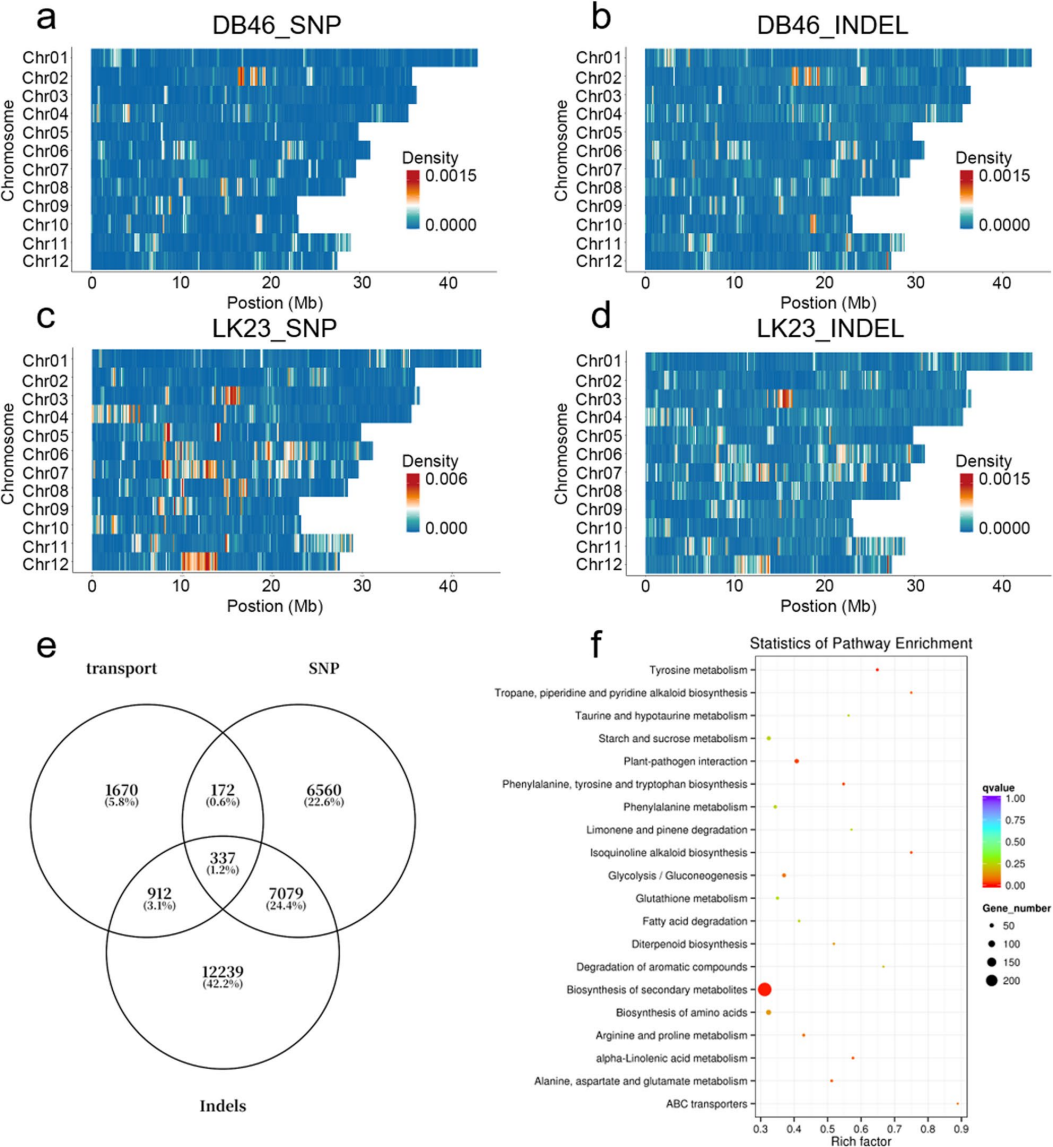
Genomic variation landscape and functional annotation of resequencing data for rice varieties DB46 and LK23. **a**–**d** Chromosomal distribution of SNP and InDel densities across the 12 rice chromosomes. The heatmaps display the density of (**a**) SNPs in DB46, (**b**) InDels in DB46, (**c**) SNPs in LK23, and (**d**) InDels in LK23. The horizontal axis represents the physical position (Mb), and the color gradient indicates variant density, ranging from blue (low density) to red (high density). (**e**) Venn diagram illustrating the intersection of genes containing SNPs, genes containing InDels, and Differentially expressed genes (DEGs) identified from the transcriptome. The numbers and percentages indicate the counts of unique and shared genes within each category. (**f**) KEGG pathway enrichment analysis of the shared genes. The y-axis lists the top enriched pathways, while the x-axis represents the rich factor. The dot size corresponds to the number of genes involved, and the color scale indicates the significance level (*Q*-value)

To compare the genomic variation patterns between the drought-tolerant cultivar DB46 and the drought-sensitive cultivar LK23, we examined the genome-wide distribution of SNPs and InDels across all 12 rice chromosomes (Fig. 5). The SNP density map revealed widespread but uneven polymorphism across the genomes of both cultivars. In DB46, SNPs were relatively evenly distributed, with moderate enrichment near Chr03 (18–20 Mb) and Chr04 (~ 15 Mb), indicating a conserved genomic background with fewer accumulated mutations, consistent with a relatively stable evolutionary state. In contrast, LK23 exhibited a substantially higher SNP density, with prominent hotspots on Chr02 (~ 10 Mb), Chr05 (15–25 Mb), Chr07 (5–15 Mb), and Chr12 (0–5 Mb). These regions likely harbor elevated local polymorphism or recombination activity, which may be associated with its weaker drought tolerance. Similarly, the InDel distribution pattern also revealed pronounced differences between the two genotypes. In DB46, InDels were evenly distributed across most chromosomes, with only slight enrichment at Chr03 (~ 18 Mb) and Chr09 (~ 20 Mb), reflecting a stable genomic structure and low structural polymorphism. Conversely, LK23 displayed higher and more uneven InDel density, with marked enrichment in Chr03 (18–20 Mb), Chr05 (10–15 Mb), and Chr12 (0–5 Mb), suggesting increased structural variation or localized genomic instability. To further identify potential drought-responsive genes, we integrated the differentially expressed genes (DEGs) from the root transcriptome with genes harboring SNP and InDel variations, resulting in 337 key candidate genes (Fig. 5e). KEGG enrichment analysis of these candidates showed significant overrepresentation of the fatty acid degradation and α-linolenic acid metabolism pathways (Fig. 5f). Notably, among these genes, 4-amino-4-deoxychorismate lyase (LOC_Os05g15530), protein phosphatase 1 L (LOC_Os06g48300), threonine phosphatase 2 C (LOC_Os09g14540), and lipoxygenase (LOC_Os12g37260) exhibited pronounced differences between the two cultivars and were consistently expressed at significantly higher levels in both roots and leaves of the drought-tolerant genotype compared with the drought-sensitive genotype. These genes have been previously reported to participate in drought stress responses in rice, highlighting their potential regulatory roles in drought adaptation mechanisms. We performed qRT–PCR validation of the above-mentioned genes, and the results were highly consistent with the transcriptome data (Figure S4), further confirming the reliability of the transcriptional changes.

## Discussion

We employed a combined strategy of “laboratory preliminary screening and field validation” to successfully identify two contrasting genotypes from 200 rice accessions: the drought-tolerant DB46 and the drought-sensitive LK23. While laboratory simulations allow for rapid high-throughput screening, the complexity of field conditions remains the ultimate standard for verifying drought tolerance. Our field trials demonstrated that under upland (drought) conditions, the yield of the sensitive genotype LK23 was severely compromised, with a precipitous decline in yield per plant. In stark contrast, DB46 exhibited superior yield stability, underscoring its potential as a valuable donor parent for breeding drought-resilient rice, particularly for ensuring stable production in arid regions. Leaves are the primary organs for transpiration and photosynthesis, and their morphological plasticity represents the most direct response to drought [35]. Typically, rice plants reduce transpiration rates through leaf rolling or reducing leaf area, a classic “drought avoidance” mechanism [36]. However, we observed a striking phenomenon: DB46 exhibited a significant increase in leaf width under drought, whereas LK23 showed a significant decrease. While the reduced leaf width and rolling in LK23 minimized water loss in the short term, it substantially restricted photosynthetic area, leading to insufficient biomass accumulation and reduced yield. Conversely, the increased leaf width in DB46 suggests a more active adaptive strategy. Leaf morphology is an important determinant of plant responses to drought stress. Previous studies have shown that leaf width and size can influence boundary layer properties, transpiration rates, and leaf temperature regulation, thereby affecting water use efficiency and stress adaptation [37]. Although reduced leaf area is often associated with drought avoidance, larger or wider leaves may confer advantages under certain conditions by maintaining photosynthetic capacity and carbon assimilation, particularly when supported by efficient hydraulic conductance and stomatal regulation [38]. Therefore, the increased leaf width observed in DB46 may contribute to drought tolerance through coordinated regulation of water transport and photosynthetic performance, although this relationship likely depends on integrated physiological traits.

Transcriptomic analysis provided molecular insights into the observed phenotypic divergence. Principal Component Analysis (PCA) revealed distinct separation between shoot and root tissues. Notably, the highest number of differentially expressed genes (DEGs) was detected in roots under upland conditions, highlighting the root system’s critical role as the primary organ for sensing and responding to soil water deficit. K-means clustering revealed distinct gene expression patterns governed primarily by tissue type and genotype rather than the external environment. For instance, Clusters 1, 3, and 4 exhibited expression patterns largely independent of both genetic background and environmental conditions, whereas Cluster 2 showed a strong genotype-dependent pattern. Compared with LK23, genes in Cluster 2 were generally suppressed in the drought-tolerant cultivar DB46. Functional enrichment analysis revealed that these genes were primarily associated with energy metabolism pathways, suggesting their involvement in stress response processes. This pattern implies that DB46 may enhance its adaptability to adverse environments by finely regulating these metabolism-related genes to optimize the synthesis and allocation of downstream metabolites. In the drought-tolerant DB46, upregulated DEGs in roots were significantly enriched in pathways including carbon metabolism, fatty acid degradation, α-linolenic acid metabolism, peroxisome metabolism, and aromatic amino acid metabolism (e.g., tryptophan and tyrosine). These pathways function synergistically to provide a continuous energy source and promote the synthesis of signaling molecules (such as jasmonic acid and its derivatives), thereby activating a cascade of stress responses. Furthermore, the enrichment of fatty acid degradation and peroxisome pathways in DB46 roots indicates a superior capacity for lipid remodeling, allowing for the rapid mobilization of stored energy via β-oxidation to maintain membrane stability and regulate redox balance. Peroxisomes serve as pivotal organelles in orchestrating cellular redox homeostasis. Through the β-oxidation pathway, they not only facilitate the rapid mobilization of stored lipids to meet energy demands under stress but also synergistically mitigate lipid peroxidation via their endogenous antioxidant systems. This dual functionality is crucial for maintaining biomembrane structural integrity and precisely regulating the intracellular redox balance [39]. Conversely, in LK23 roots, these same metabolic pathways were significantly downregulated, suggesting limited energy generation and signal transduction efficiency, leading to a delayed or suppressed stress response. GO enrichment analysis further supported this: genes related to monooxygenase and oxidoreductase activity were significantly upregulated in DB46 (97 and 125 genes, respectively) but downregulated in LK23, accompanied by the downregulation of 10 dioxygenase-related genes. Monooxygenases and oxidoreductases are key components of plant redox metabolism and play important roles in drought stress responses. Monooxygenases, particularly members of the cytochrome P450 and flavin-containing monooxygenase (FMO) families, participate in the biosynthesis of hormones and secondary metabolites that are involved in stress signaling and adaptation. For example, cytochrome P450 monooxygenases are widely implicated in the production of protective metabolites and stress-related signaling molecules under abiotic stress conditions, including drought [40]. In addition, flavin-containing monooxygenases such as YUCCA proteins contribute to drought tolerance by regulating reactive oxygen species (ROS) homeostasis and maintaining cellular redox balance [41]. As core components of the antioxidant defense system, oxidoreductases play a pivotal role in scavenging drought-induced reactive oxygen species (ROS) and maintaining cellular redox homeostasis [42]. This discrepancy indicates that DB46 possesses a more robust capacity for reactive oxygen species (ROS) scavenging, lipid oxidative modification, and metabolic reallocation, mitigating membrane lipid peroxidation and maintaining metabolic homeostasis under water deficit.

Metabolomic data corroborated the transcriptional trends. KEGG enrichment analysis showed that root metabolites in DB46 were significantly enriched in fatty acid biosynthesis, metabolism, and degradation, as well as phenylalanine and flavonoid biosynthesis pathways. The enrichment of lipid and secondary metabolism pathways in DB46 under upland conditions was far superior to that of LK23, indicating an enhanced adaptive mechanism through lipid remodeling and secondary metabolite networks. Specifically, 12 lipid-related metabolites significantly accumulated in DB46 roots under drought, whereas they significantly decreased in LK23. In contrast, lipid metabolic changes in leaves were less pronounced; DB46 maintained relative stability, while LK23 showed a reduction in various lipid metabolites. This underscores the root system as the central metabolic regulatory hub for drought response. Fatty acid biosynthesis not only provides essential precursors for cuticular wax deposition to minimize non-stomatal water loss, but the activation of fatty acid degradation (β-oxidation) also facilitates energy mobilization to fuel various stress-responsive processes [43]. Collectively, DB46 utilizes dual transcriptional and metabolic regulation to activate fatty acid degradation, α-linolenic acid metabolism, and ROS detoxification pathways, ensuring the coordinated maintenance of energy supply, signal transduction, and membrane integrity. LK23, hampered by gene downregulation and metabolic lag, suffered from inefficient lipid metabolism and excessive ROS accumulation, resulting in oxidative stress and physiological damage.

The integration of transcriptomics and metabolomics provided the most compelling evidence for the molecular divergence between the two genotypes. In DB46 roots, the fatty acid β-oxidation pathway appeared highly active, characterized by the significant accumulation of specific free fatty acids (e.g., decanoic acid) and acyl-CoA intermediates (e.g., butanoyl-CoA), despite the transcriptional downregulation of key enzyme genes (e.g., *ACSL*,* ACOX*,* ACADM*). This “uncoupling” between transcription and metabolism, where metabolite accumulation occurs despite reduced gene expression, suggests that DB46 maintains metabolic balance through efficient post-transcriptional regulation or rapid metabolic flux, preventing the toxic over-accumulation of intermediates. Similar discrepancies between transcript abundance and metabolite accumulation have been widely reported in plant stress responses, reflecting the multi-layered and dynamic regulation of plant metabolism, which is influenced not only by transcriptional control but also by enzyme activity, substrate availability, and metabolic flux [44, 45]. The net result is enhanced lipid mobilization and energy reallocation, crucial for maintaining cellular function and osmotic homeostasis. In sharp contrast, LK23 exhibited an opposing pattern: general upregulation of β-oxidation genes but a marked decrease in free fatty acids and acyl-CoA intermediates. This points to a low coupling efficiency between transcription and metabolism, indicating restricted fatty acid processing and insufficient lipid mobilization to power drought adaptation. Additionally, the enrichment of secondary metabolite biosynthesis (flavonoids and phenylpropanoids) in DB46 roots highlights a stronger defense synthesis capability. These compounds are widely recognized for their antioxidant and cytoprotective roles, effectively mitigating drought-induced oxidative damage [46].

Whole-genome resequencing (WGS) offered critical clues regarding the genetic basis of the observed drought responses. The higher level of genomic variation in LK23 suggests a more dynamic but structurally less stable genome, which may correlate with its poor drought tolerance. Conversely, DB46 displayed moderate and evenly distributed polymorphism, reflecting a conserved genomic background where long-term selection may have fixed favorable alleles related to drought resistance. The SNP density map revealed significant differences: DB46 showed a relatively even distribution across the 12 chromosomes with minor enrichment near Chr03 and Chr04, indicating fewer recombination hotspots and a stable genetic structure. LK23, however, exhibited distinct high-density hotspots of SNPs and InDels on Chr02, Chr05, Chr07, and Chr12. These regions of high polymorphism likely represent areas of intense local recombination or mutation, potentially harboring deleterious variations that undermine stress adaptation. By integrating transcriptomic data with genomic variations, we identified 337 candidate genes that exhibited both significant differential expression and SNP/InDel variations. KEGG analysis revealed that these genes were predominantly enriched in fatty acid degradation and α-linolenic acid metabolism pathways, consistent with our omics findings. These pathways are pivotal for membrane lipid remodeling, energy homeostasis, and the synthesis of signaling molecules like jasmonic acid. Notably, several key genes, including *4-amino-4-deoxychorismate lyase* (*LOC_Os05g15530*), *protein phosphatase 1 L* (*LOC_Os06g48300*), *threonine phosphatase 2 C* (*LOC_Os09g14540*), and *lipoxygenase* (*LOC_Os12g37260*), showed pronounced differences in both sequence and expression levels between the two cultivars. These genes are established regulators of drought response in rice, involved in redox balance, lipid peroxidation, and hormonal signaling [47–49]. For instance, lipoxygenase catalyzes the oxidation of polyunsaturated fatty acids to generate oxylipins, triggering stress signaling cascades, while PP1L and PP2C are integral components of ABA and MAPK signaling pathways regulating stomatal dynamics [50]. In summary, DB46 possesses a stable genomic architecture enriched with favorable allelic variations related to lipid remodeling and hormonal signaling, conferring superior drought adaptability. In contrast, the excessive genomic polymorphism in LK23 likely disrupts the coordinated regulation of these pathways, leading to delayed physiological responses and reduced tolerance. This study establishes a systematic molecular framework linking genomic variation to metabolic and transcriptional regulation, providing key targets for future molecular breeding.

## Conclusion

Through integrated analyses of field phenotyping, transcriptomics, metabolomics, and whole-genome resequencing, this study systematically elucidates the divergent drought-response mechanisms between rice cultivars DB46 and LK23 (Figure S5). Field evaluations demonstrated that DB46 maintained superior yield stability and exhibited a positive morphological adaptation, an increase in leaf width, under upland conditions, whereas LK23 showed pronounced leaf rolling and narrowing that temporarily reduced transpiration but markedly limited photosynthetic capacity and biomass accumulation. At the molecular level, DB46 roots displayed coordinated up-regulation of pathways related to fatty acid degradation, α-linolenic acid metabolism, and reactive oxygen species (ROS) detoxification, enabling efficient energy mobilization, membrane stabilization, and redox homeostasis. In contrast, these pathways were broadly down-regulated in LK23, resulting in insufficient energy supply and excessive oxidative stress. Metabolomic profiling further confirmed that DB46 exhibited stronger enrichment in lipid and secondary-metabolism pathways, including fatty acid biosynthesis, fatty acid metabolism, fatty acid elongation, fatty acid degradation, flavonoid biosynthesis, and phenylalanine metabolism, forming a robust antioxidant defense system. Genome-wide variation analysis revealed that DB46 possesses a more stable and conserved genomic structure, whereas LK23 shows higher local polymorphism and structural variation, which may weaken its regulatory coordination under stress. Integrated genomic and transcriptomic analyses identified 337 candidate genes associated with lipid metabolism and redox regulation, including *lipoxygenase*, *protein phosphatase 1 L*, and *PP2C*, that are likely key molecular determinants of drought adaptation. Overall, DB46 achieves efficient drought tolerance and yield stability through the synergistic integration of morphological plasticity, metabolic flexibility, and genomic stability, forming a comprehensive “lipid–redox” regulatory network. These findings not only clarify the molecular basis of drought resilience in rice but also provide essential genetic targets and theoretical guidance for molecular design breeding aimed at improving drought tolerance and stable productivity in water-limited agroecosystems. 

## Supplementary Information

Supplementary Material 1.

Supplementary Material 2.

## Data Availability

The datasets generated and/or analysed during this study have been deposited in the Sequence Read Archive (SRA) database of the National Center for Biotechnology Information and the Genome Sequence Archive (GSA) database under BioProject accession numbers PRJNA1418826 and CRA038395, respectively. The corresponding links are https://www.ncbi.nlm.nih.gov/sra/?term=PRJNA1418826 and https://ngdc.cncb.ac.cn/gsa/browse/CRA038395.
