# Supplementary material for: Integrated multi-omics analysis reveals a lipid–redox regulatory network underlying drought tolerance and yield stability in rice
Source: BMC Plant Biol. 2026 May 27;26:1250. doi: 10.1186/s12870-026-09098-1 (PMC13397616; doi:10.1186/s12870-026-09098-1)

Figure S1. Comparison of grain yield per plant between DB46 and LK23 under paddy and upland conditions.


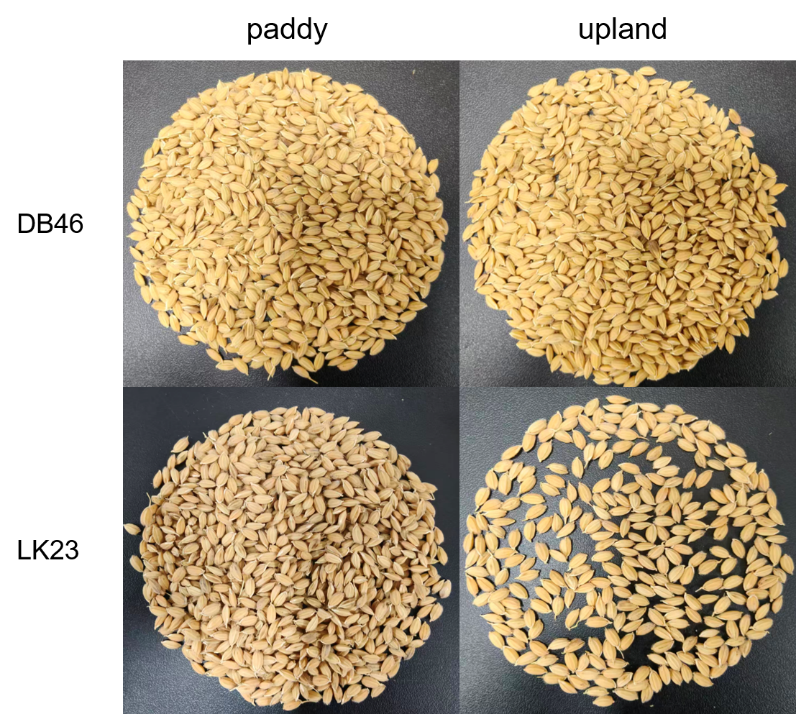


Figure S2. Gene Ontology (GO) enrichment analysis of differentially expressed genes (DEGs) responding to upland conditions in the roots of DB46 and LK23. (a) Significantly enriched GO terms in the DBp_vs_DBu comparison group. (b) Significantly enriched GO terms in the LDp_vs_LDu comparison group. The terms are classified into three main categories: Biological Process (BP, blue bars), Cellular Component (CC, green bars), and Molecular Function (MF, orange bars). The y-axis represents the significance of enrichment, expressed as -log10 (adjusted *P*-value). The numbers above the bars indicate the number of DEGs enriched in each specific term.


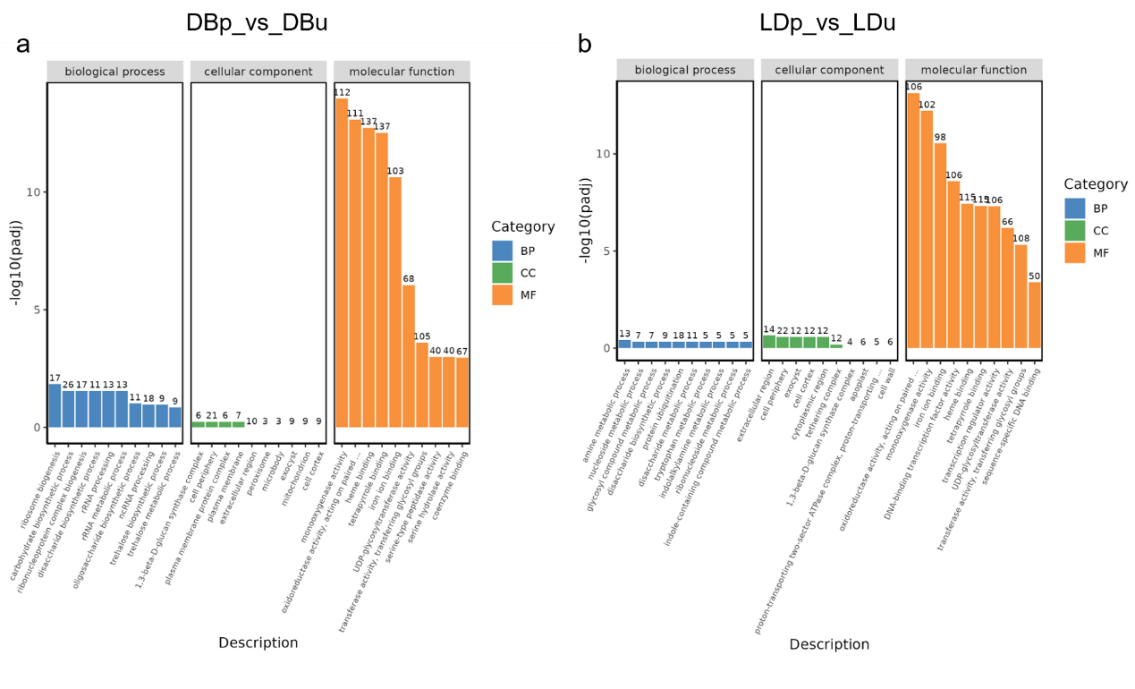


Figure S3. Integrated KEGG pathway enrichment analysis of differentially expressed genes (DEGs) and differentially accumulated metabolites (DAMs) under upland conditions. (a) Top enriched KEGG pathways in the DB46 variety (paddy vs. upland comparison). (b) Top enriched KEGG pathways in the LK23 variety (paddy vs. upland comparison). The y-axis lists the enriched pathways. The x-axis represents the enrichment ratio (Rich factor), calculated as the ratio of the number of DEGs/DAMs to the total number of annotated genes/metabolites in the pathway. The size of the dots corresponds to the count of mapped DEGs and DAMs, while the color gradient indicates the significance level (*P*-value), ranging from blue (higher *P*-value) to red (lower *P*-value).


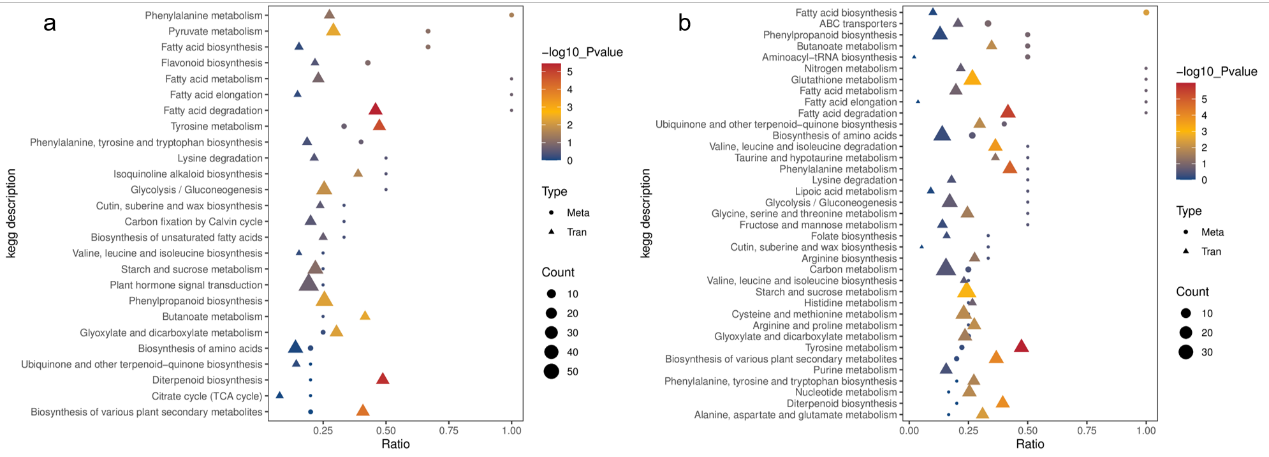


Figure S4. Gene expression levels in the roots of two rice cultivars under different cultivation conditions were validated using qRT-PCR, asterisks indicate statistical significance by two-tailed Student’s t tests (**P* < 0.05, ***P* < 0.01).


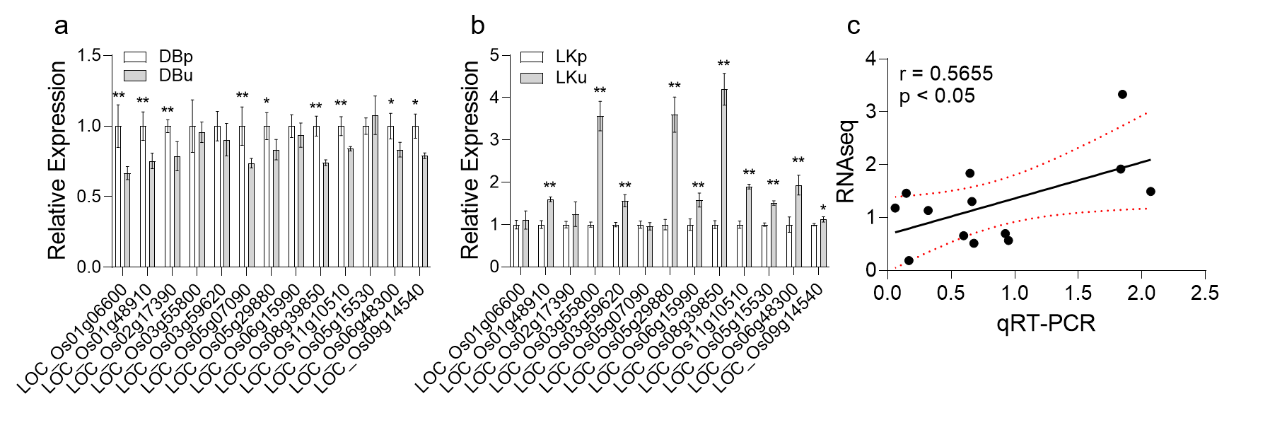


Figure S5. Multi-omics workflow summarizing the study design.


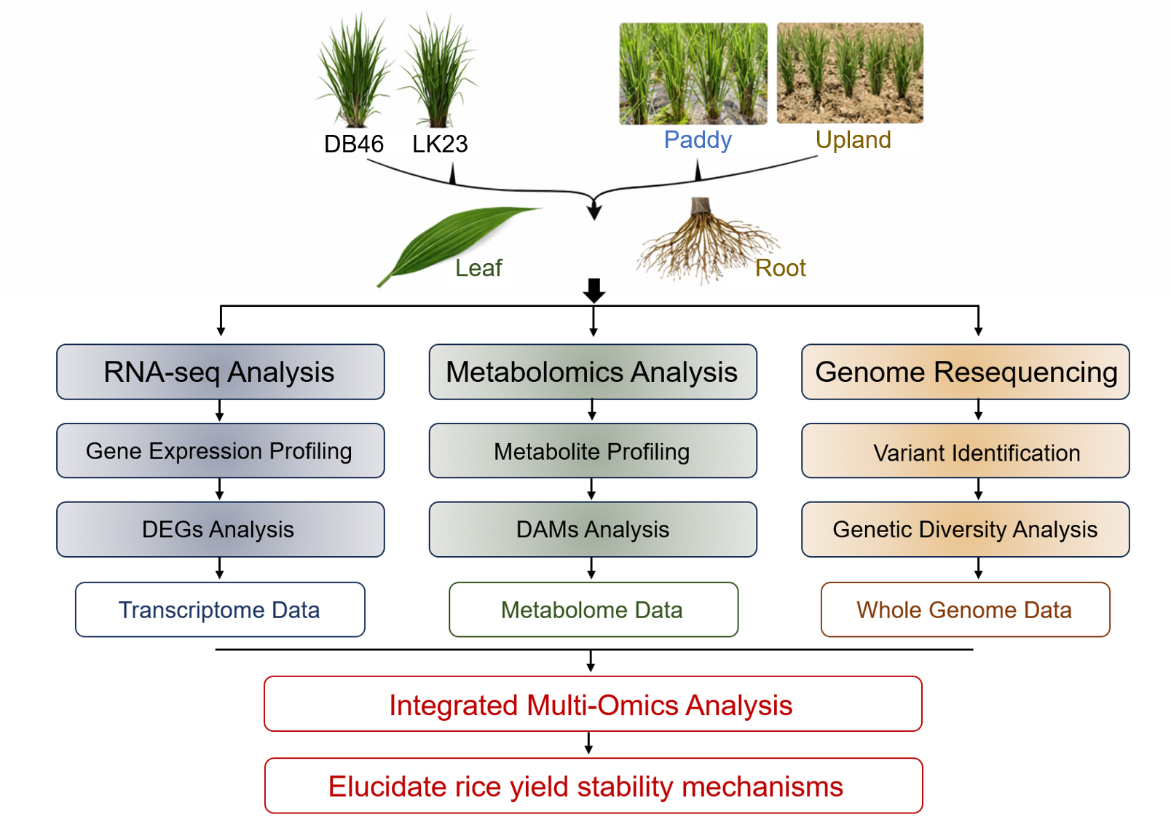

Supplement: Supplementary file 1 — Supplementary Material 1. [file 12870_2026_9098_MOESM1_ESM.docx]
